# 

**Published:** 1836-04

**Authors:** 


### CONTENTS OF No. II.

OF THE

### BRITISH AND FOREIGN MEDICAL REVIEW.

### Part First.—Analytical and Critical Reviews.

Art. I.—1. The Philosophy of Health; or, an Exposition of the Physical and Mental Constitution of Man, with a View to the Promotion of Human Longevity and Happiness. By Southwood Smith, m.d. (*With Woodcuts*) . . 313

2. The Principles of Physiology applied to the Preservation of Health and to the Improvement of Physical and Mental Education. By Andrew Combe, m.d. 328

3. On the Influence of Atmosphere and Locality; Change of Air and Climate; Seasons; Food; Clothing; Bathing; Exercise; Sleep; Corporeal and Intellectual Pursuits, &c. on Human Health; constituting Elements of Hygiène. By Robley Dunglison, m.d. . . . . . 344

4. Lectures on the Ordinary Agents of Life, as applicable to Therapeutics and Hygiène; or, the Uses of the Atmosphere, Habitations, Baths, Clothing, Climate, Exercise, Food, Drinks, &c. in the Treatment and Prevention of Disease. By Alexander Kilgour, m.d. . . . . 352

5. Lectures on the Means of Promoting and Preserving Health, delivered at the Mechanic's Institute, Spitalfields. By T. Hodgkin, m.d. . . 362

Art. II.—Report of the Fourth Meeting of the British Association for the Advancement of Science; held at Edinburgh, in 1834 . . . 368

1. Report of the State of our Knowledge of the Laws of Contagion. By Wm. Henry, m.d. f.r.s. &c. . . . . 369

2. Report on Animal Physiology; comprising a Review of the Progress and State of Theory, and of our Information respecting the Blood and the Powers which circulate it. By Wm. Clark, m.d. f.r.c. &c. . . . 379

Art. III.—1. An Essay on Clinical Instruction, by P. Ch. A. Louis, m.d. Translated by Peter Martin, m.r.c.s. . . . . 397

2. Recherches sur les Effets de la Saignée dans quelques Maladies Inflammatoires, et sur l'Action de l'Emétique et des Vésicatoires dans la Pneumonie. Par P. Ch. A. Louis, m.d. . . . . . 405

3. De l'Emploi du Tartre Stibié à haute Dose dans le Traitement des Maladies en général, et dans celui de la Pneumonie et du Rheumatisme en particulier. Par Professeur Alm. Lepelletier de la Sarthe . . 415

Art. IV.—Traité Clinique des Maladies du Cœur, precédé de Recherches nouvelles sur l'Anatomie et la Physiologie de cet Organe. Par Professeur Bouillaud . 424

Art. V.—Practical Observations on Diseases of the Heart, Lungs, Stomach, Liver, &c., occasioned by Spinal Irritation; and on the Nervous System in general as a Source of Organic Disease. By John Marshall, m.d. . . 459

Art. VI.—Ueber den Markschwamm der Hoden. Vom Dr. Otto Baring . 469

### Part Second.—Bibliographical Notices.

Art. I.—Nouveau Manuel complet d'Auscultation et de Percussion, ou Application de l'Acoustique au Diagnostic des Maladies. Par M. Raciborski, m.d.p. . 479
Art. II.—An Introduction to Hospital Practice, in various Complaints; being a Clinical Report of Fever, Gout, Rheumatism, Cholera, Jaundice, Erysipelas, Insanity, &c., and Diseases of the Chest and Heart; with Remarks on their Pathology and Treatment. By C. J. B. Aldis, m.a. m.b. &c. . . 490
Art. III.—Observations on the principal Medical Institutions and Practice of France, Italy, and Germany; with Notices of the Universities, and Cases from Hospital Practice. To which is added an Appendix, on Animal Magnetism and Homœopathy. By Edwin Lee, m.r.c.s. . . . . 494
Art. IV.—Commentatio Medico-Practica de Morbis Intestini Cæci, et de dignitate hujus Visceris Pathologica in dijudicanda Passione Colica et Iliaca. By Dr. Unger 501
On Phlegmonous Tumours in the Right Iliac Region. By J. M. Ferrall, Esq. ib.
Observations upon a peculiar Disease of the Cæcum, or Caput Coli; contained in a Letter to Dr. Crampton, from Francis W. Smith, m.d. . . ib.
Art. V.—Observations on the Climate, Soil, and Productions of British Guiana, &c.; with Remarks on the Diseases, their Treatment and Prevention, founded on a long Experience within the Tropics. By John Hancock, m.d. . 508
Art. VI.—Account of the Discovery, by Purkinje and Valentin, of Ciliary Motions in Reptiles and Warm-blooded Animals. With Remarks and additional Experiments. By William Sharpey, m.d. f.r.s. &c. . . . 509
Art. VII.—Manual of Practical Midwifery; containing a Description of Natural and Difficult Labours, with their Management. Intended chiefly as a book of Reference for Students and junior Practitioners. By James Reid, m.d. &c. . 512
Art. VIII.—A Manual of Select Medical Bibliography, in which the Books are arranged chronologically according to the Subjects, and the Derivations of the Terms and the nosological and vernacular Synonyms of the Diseases are given. With an Appendix, containing Lists of the collected Works of Authors, Systematic Treatises on Medicine, Transactions of Societies, Journals, &c. By John Forbes, m.d. f.r.s. . . . . . 513
Art. IX.—A Dictionary of Terms used in Medicine and the Collateral Sciences. By Richard Hoblyn, m.a. . . . . . . 517
Art. X.—The Medical Student's Practical and Theoretical Guide to the Translation and Composition of Latin Prescriptions; with an explanatory Latin Grammar, written in a familiar Style; together with a Text-book and Exercises, calculated to make the Learner thoroughly acquainted with the verbal and grammatical Analysis of the Pharmacopœia, Prescriptions, &c. By J. W. Underwood . . . . . . 519
Art. XI.—A Literal Interlineal Translation of the first Ten Chapters of Gregory's Conspectus Medicinæ Theoreticæ; with the original Text. To which are added, an Ordo Verborum, and Rules for Construing, &c. By R. Venables, a.m. m.b. 520
Art. XII.—De Glandularum Intestinalium Structura Penitiori. Commentatio Anatomica scripsit Dr. Ludovicus Boehm. (*With a Lithographic Plate.*) . 521
Art. XIII.—Transactions of the Provincial Medical and Surgical Association . 529
Art. XIV.—Observations on the Action of the Broom-Seed in Dropsical Affections. By Richard Pearson, m.d. . . . . 533
Art. XV.—On Perforation and Division of Permanent Stricture of the Urethra by the Lancetted Stilettes; with Observations on the Nature and Treatment of Spasmodic and Inflammatory Stricture, and in various other Urethral Affections. By R. A. Stafford, Esq. Third Edition . . . . 534
Art. XVI.—A Treatise on Hydrocephalus, or Water on the Brain; with the most successful Modes of Treatment. By Wm. Griffith, Esq. . . 535
Art. XVII.—The Cyclopædia of Anatomy and Physiology. Edited by Robert B. Todd, m.b. Parts I. to IV. . . . . . 536

ART. XVIII.—Practical Anatomy of the Nerves and Vessels supplying the Head, Neck, and Chest: intended as a Guide for the Use of Students in the Dissection of those Structures. By E. COCK, Esq. . . . 541
ART. XIX.—Guy's Hospital Reports. No. I. January, 1836. Edited by GEORGE H. BARLOW, M.A. and JAMES P. BABINGTON, M.A. . . . 543
St. Thomas's Hospital Reports. By JOHN F. SOUTH, Assistant Surgeon. No. II. January, 1836 . . . . . ib.
ART. XX.— THE FOREIGN JOURNALS.
THE GERMAN JOURNALS:
7. Magazin für die gesammte Heilkunde. Herausgegeben von J. N. RUST 545
8. Neue Zeitschrift für Geburtskunde. Herausgegehen von BUSCH, D'OUTREPONT, und RITGEN . . . . ib.
9. Zeitschrift für die gesammte Medicin mit besonderer rücksicht auf hospitalpraxis und ausländische Literatur. Herausgegeben von J. F. DIEFFENBACH, J. C. G. FRICKE, und F. W. OPPENHEIM . 546
ITALIAN JOURNALS:
4. L'Eclettico di Adone Palmierj, Giornale di Medicina, Chirurgia, Economia Domestica, Arte Industriale, Agricoltura, &c. . ib.
5. Bullettino delle Scienze Mediche, pubblicato per cura della Società Medico-Chirurgica di Bologna, e redatto dai Socj Prof. BARONI, &c. 547
FRENCH JOURNALS:
6. Journal Hebdomadaire des Progrès des Sciences et Institutions Médicales, ib.
AMERICAN JOURNALS:
3. The United States Medical and Surgical Journal . . 548

---

### PART THIRD.—Selections from Foreign Journals.

### ANATOMY.

On the Structure of Bones. By MICHELE MEDICI, M.D. . . . 549
Of the supposed Origin of the Posterior Root of the first Cervical from the eleventh Cerebral (Accessory) Nerve. By FR. ARNOLD . . 550
Of the Development of the Iris. By FR. ARNOLD . . . 551

### PHYSIOLOGY.

Experiments on Saliva. By Dr. C. H. SCHULTZ . . . ib.
On the Respiration and Pulse of the Old. By MM. HOURMANN and DECHAMBRE 553
Experiments on the Brain, Spinal Marrow, and Nerves. (*With Woodcuts.*) By Professor MAYER, of Bonn . . . . . 556

### MEDICINE,

#### PATHOLOGICAL, PRACTICAL, AND THERAPEUTICAL.

On Diseases of the Spleen, as they occur in India. By J. O. VOIGT, M.D. . 563
On the Use of the Ballota Lanata (Linn.) in the cure of Rheumatic, Arthritic, and Gouty Affections. By Professor V. L. BRERA . . . 566
Alum in Typhoid Fevers. By Professor FOUQUIER . . . 568
On the Treatment of Croup by Sulphate of Copper. By K. G. ZIMMERMAN, M.D. ib.
On Turpentine in Sciatica. By M. DUCROS, Jun. . . . 569
On Purulent Metastasis. By Dr. R. FRORIEP . . . ib.
On the proper Temperature of Sinapisms . . . . 570
On Salicine as a Substitute for Quinine. By Dr. PLEISCHL . . ib.
Case of Poisoning by Arsenic, successfully treated by the hydrated Tritoxide of Iron. By M. GEOFFROY . . . . . 572
Hydrated Peroxide of Iron as an Antidote to Arsenic. By MM. BINEAU and MAJESTÉ, 573
Fumigations in Hooping-Cough. By Dr. DOHM . . . 574

### SURGERY.

On the Nature and Treatment of the Vertebral Disease of Pott. By M. Ri het 574
Treatment of Scald Head, (Teigne, Fr. Porrigo.) By Baron Alibert . 578
On Balsam of Copaiba in Gonorrhœa. By M. Ratier . . . ib.
On the Use of the Datura Stramonium, in Sarcocele and Diseases of a similar character. By Thomas T. Everitt, m.d. . . ib.
Ointment to allay the Irritation of Hemorrhoidal Tumours. By Dr. Geddings 579
On a new Mode of Treating Burns. By Professor Velpeau . . ib.
On the Treatment of Burns with Yellow Wash, (Aqua Phagædenica.) By F. B. E. Hintze, m.d. . . . . . 581
Case of Viper's Bite. By Dr. Siebergundi, of Dorsten . . 582
Hernia of the Appendix Cæci Vermiformis. By Dr. Taramelli . . 583
On the Treatment to be adopted after the Reduction of Dislocations. By Professor Malgaigne . . . . . . . ib.
New Mode of Treating Luxations of the Sternal End of the Clavicle. By M. Velpeau 584
On the Seat and Diagnosis of Dislocations of the Shoulder-Joints. By M. Malgaigne 585
Observations on the Employment of the Suction Pump in Incarcerated Hernia. By Dr. L. Kochler . . . . . . 586

### MIDWIFERY.

On the Reposition of the Umbilical Cord. By Dr. Michaelis . . 588
Tables of Midwifery Cases in the Maison d'Accouchement de Paris, from 15th June, 1830, to 15th June, 1835 . . . . 589

### ANIMAL CHEMISTRY.

Researches on the Blood. By L. Gmelin, F. Tiedemann, and E. Mitscherlich 590

### MEDICAL JURISPRUDENCE AND TOXICOLOGY.

Experiments on Peroxide of the Hydrate of Iron as a Counter-poison to Arsenic. First Series. By MM. Miguel and Soubeiran . . . 594
Second Series. By Drs. G. Borelli and C. Demaria . . . 595
On the Means of Preventing Poison. By MM. Chevalier and Boys de Loury 596

---

### Part Fourth.—Medical Intelligence.

A Sketch of the Origin and Present State of Medicine, and of Medical Institutions in Russia. By George Lefevre, m.d., late Physician to the British Embassy at St. Petersburg, &c.—Part I. *Introductory* . . 597
Medical Association of Belgium . . . . . 606
Medical Reform in Spain . . . . . . 608
Qualification of Dentists in Prussia . . . . . ib.
Encouragement of Vaccination in Prussia . . . . 609
Relative Value of Apothecaries' Weights in different Countries . . ib.
Irish Medical Corporations . . . . . 610
Anatomy of the Ovum, in the Ovary. By Thomas Wharton Jones, Esq. . ib.
OBITUARY.—Sir David Barry, m.d. . . . . 611
William Twining, Esq. . . . . 612
John Cheyne, m.d. f.r.s. m.r.i.a. . . . 613
Dr. Marsden . . . . . 615

List of Original Papers in the British Journals during the last Quarter . 615
List of Books received for Review . . . . . 619

